# Supplementary material for: Harnessing the flexibility of neural networks to predict dynamic theoretical parameters underlying human choice behavior
Source: PLoS Comput Biol. 2024 Jan 4;20(1):e1011678. doi: 10.1371/journal.pcbi.1011678 (PMC10793919; doi:10.1371/journal.pcbi.1011678)
Supplement: S1 Text — (PDF) [file pcbi.1011678.s001.pdf]

S1 Sensitivity of t-RNN as a function of the number of observations and free parameters in the theoretical RL model. Here, we examine the impact of the number of trials available and the number of free parameters for the theoretical RL parameters on the quality of t-RNN parameter recovery. Specifically, we compared the performance of the stationary maximum-likelihood (MLE; fitted with 5 different search locations) and the t-RNN models trained with one, two, or three theoretical free parameters. Using the Q-learning and preservation model, we systematically increased the number of free parameters (see Table A) and performed parameter recovery on 150 test agents (simulated with stable parameters). Additionally, we systematically increased the number of trials used to find optimal parameters in increments of 25. We computed the mean-square error (MSE) between the true parameters and the estimated parameters.

Results revealed that when the RL theoretical model included only one free parameter (i.e., learning rate) stationary MLE method showed better performance compared with t-RNN. However, when we included two or three free theoretical parameters, t-RNN did not fall behind the MLE approach. Importantly, we found preliminary results suggesting that for agents with more than one free parameter, t-RNN might outperform the MLE approach when the data has a very low number of observations per individual (∼25 to 50). Furthermore, a close examination of the results shows that the MLE method for models with more than one parameter showed somewhat less consistent improvement in MSE as a function of trials compared with t-RNN (see Fig A). This is most likely the result of MLE being more sensitive to initial values (we used five starting points that were chosen randomly for each agent, and used for the results the starting values that gave the best result). Overall, t-RNN has the advantage of being able to predict dynamic parameters across trials, and the current analysis adds that when the thermotical parameters are stable, t-RNN does not fall behind conventional MLE approach and might even outperform MLE when the number of observations is limited.

Table A. Experiment specification.

Exp. 1	$\alpha \sim U(0,1)$	$\beta = 3$ (fix)	$\kappa = 0$ (fix)
Exp. 2	$\alpha \sim U(0,1)$	$\beta \sim U(0,10)$	$\kappa = 0$ (fix)
Exp. 3	$\alpha \sim U(0,1)$	$\beta \sim U(0,10)$	$\kappa \sim U(-.5,.5)$

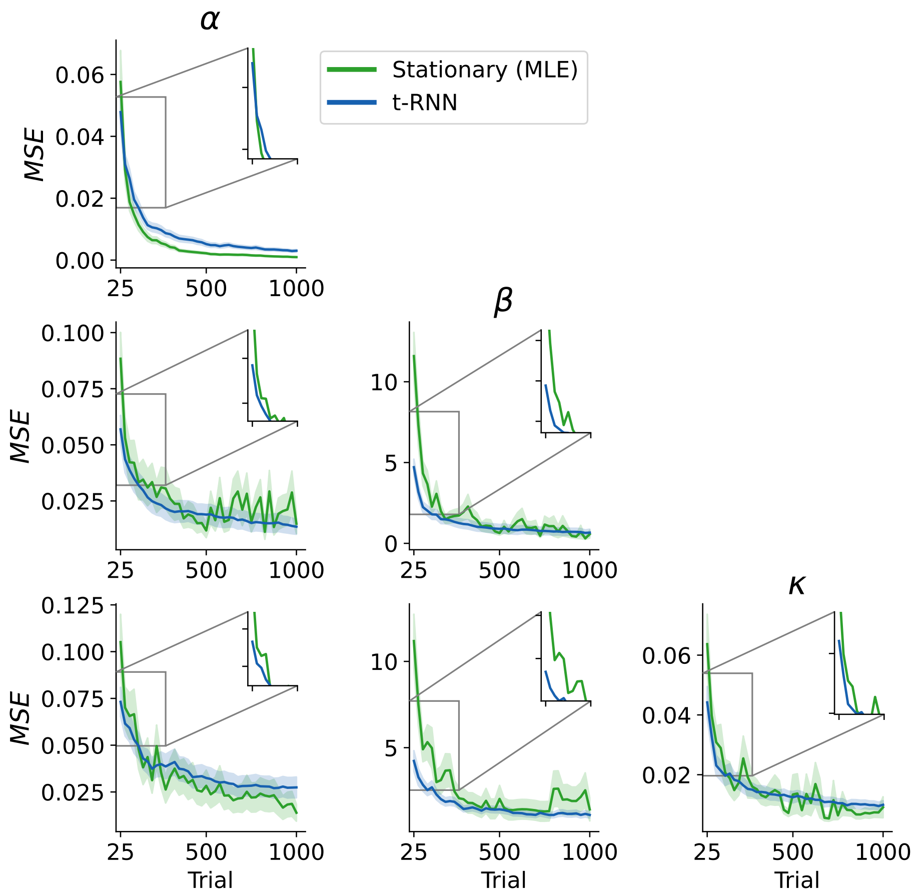

Fig A. Sensitivity to the trial numbers and free parameters. Parameter recovery performance (measured in MSE and averaged over 150 test agents; y-axis) for the stationary (maximum-likelihood; green) and t-RNN (blue) as a function of available trials (x-axis). We also vary the number of free parameters, represented by: the top, middle, and bottom panels for Exp. 1, Exp. 2, and Exp. 3, respectively (shaded area signifies s.e.m; top right corner zoom in on first 100 trials).
