# Supplementary material for: Harnessing the flexibility of neural networks to predict dynamic theoretical parameters underlying human choice behavior
Source: PLoS Comput Biol. 2024 Jan 4;20(1):e1011678. doi: 10.1371/journal.pcbi.1011678 (PMC10793919; doi:10.1371/journal.pcbi.1011678)
Supplement: S1 Table — To ensure that quantization did not affect our performance, we ran two experiments where we quantized the RL parameters to as low as 3 bins (α and β parameters to 3 evenly spaced bins each) and up to 10 bins (α and β parameters to 10 evenly spaced bins each). We found similar performance across all three quantization settings. (PDF) [file pcbi.1011678.s002.pdf]

Sensitivity of t-RNN to parameters quantization. To ensure that quantization did not affect our performance, we ran two experiments where we quantized the RL parameters to as low as 3 bins (α and β parameters to 3 evenly spaced bins each) and up to 10 bins (α and β parameters to 10 evenly spaced bins each). We found similar performance across all three quantization settings.

Table S1. Action prediction (BCE) and parameters estimation (MSE) for different sizes of quantization bins of t-RNN. Averaged across $N = 30$ artificial test-agents. $\downarrow$ Lower is better. Mean $\pm$ SD.

**Bins size**	Action (BCE $\downarrow$)	α (MSE $\downarrow$)	β (MSE $\downarrow$)
3×3	0.321$\pm$0.12	0.024$\pm$0.01	0.020$\pm$0.01
5×5 (main text)	0.321$\pm$0.12	0.024$\pm$0.01	0.019$\pm$0.01
10×10	0.322$\pm$0.13	0.025$\pm$0.01	0.019$\pm$0.01
