# Supplementary material for: Harnessing the flexibility of neural networks to predict dynamic theoretical parameters underlying human choice behavior
Source: PLoS Comput Biol. 2024 Jan 4;20(1):e1011678. doi: 10.1371/journal.pcbi.1011678 (PMC10793919; doi:10.1371/journal.pcbi.1011678)
Supplement: S2 Table — Summary table of the raw results presented in the validation study of the main text (see Fig 2A). (PDF) [file pcbi.1011678.s003.pdf]

Action prediction and RL parameter recovery of simulated test agents. Summary table of the raw results presented in the validation study of the main text (see Fig 2A).

Table S2. Action prediction (BCE) and parameters estimation (MSE) of simulated data. Averaged across $N = 30$ artificial test-agents. $\downarrow$ Lower is better. Mean $\pm$ SD.

**Model**	Action (BCE $\downarrow$)	α (MSE $\downarrow$)	β (MSE $\downarrow$)
Q-stationarity	0.350 $\pm$ 0.15	0.053 $\pm$ 0.10	0.073 $\pm$ 0.10
Bayesian (particle filter)	0.327 $\pm$ 0.13	0.031 $\pm$ 0.03	0.038 $\pm$ 0.03
t-RNN	0.321 $\pm$ 0.12	0.024 $\pm$ 0.01	0.019 $\pm$ 0.01
