# Supplementary material for: Harnessing the flexibility of neural networks to predict dynamic theoretical parameters underlying human choice behavior
Source: PLoS Comput Biol. 2024 Jan 4;20(1):e1011678. doi: 10.1371/journal.pcbi.1011678 (PMC10793919; doi:10.1371/journal.pcbi.1011678)
Supplement: S3 Table — To assess the performance of t-RNN model in a volatile environment, we conducted an additional analysis similar to the one described in the main text (see Validating t-RNN using synthetic behavior) with agents simulated in a two-armed bandit task. However, here instead of a fixed reward probability for each arm, the reward expected value schedule was governed by a random walk with a drift rate of 0.025, and upper and lower bounds of 0.15 and 0.85, respectively. Our findings align with the conclusions mentioned in the main text, demonstrating that the t-RNN outperformed the alternatives in terms of both action prediction and parameter estimation. Therefore, we conclude that our conclusions regarding tRNN performance generalized to two-armed bandit tasks in a volatile environment. (PDF) [file pcbi.1011678.s004.pdf]

Volatile environment. To assess the performance of t-RNN model in a volatile environment, we conducted an additional analysis similar to the one described in the main text (see Validating t-RNN using synthetic behavior) with agents simulated in a two-armed bandit task. However, here instead of a fixed reward probability for each arm, the reward expected value schedule was governed by a random walk with a drift rate of 0.025, and upper and lower bounds of 0.15 and 0.85, respectively. Our findings align with the conclusions mentioned in the main text, demonstrating that the t-RNN outperformed the alternatives in terms of both action prediction and parameter estimation. Therefore, we conclude that our conclusions regarding tRNN performance generalized to two-armed bandit tasks in a volatile environment.

Table S3. Action prediction (BCE) and parameters estimation (MSE) of simulated data in a volatile environment. Averaged across $N = 30$ artificial test agents. ↓ Lower is better. Mean ± SD.

**Model**	Action (BCE ↓)	α (MSE ↓)	β (MSE ↓)
Q-stationarity	0.468 ± 0.14	0.045 ± 0.05	0.053 ± 0.07
Bayesian (particle filter)	0.461 ± 0.14	0.035 ± 0.03	0.044 ± 0.05
t-RNN	0.454 ± 0.12	0.028 ± 0.01	0.030 ± 0.03
