# Supplementary material for: Harnessing the flexibility of neural networks to predict dynamic theoretical parameters underlying human choice behavior
Source: PLoS Comput Biol. 2024 Jan 4;20(1):e1011678. doi: 10.1371/journal.pcbi.1011678 (PMC10793919; doi:10.1371/journal.pcbi.1011678)
Supplement: S4 Table — Summary table of the raw results presented in the main text (see Fig 3A). (PDF) [file pcbi.1011678.s005.pdf]

Action prediction behavioral dataset [1]. Summary table of the raw results presented in the main text (see Fig 3A).

Table S4. Action prediction for each model divided by diagnostic label. Behavioral dataset from [1]. Measured with binary cross-entropy (BCE; ↓ lower is better). Mean ± SD.

**Model**	Bipolar $N = 33$	Depression $N = 34$	Healthy $N = 34$
Q-stationarity	0.418 ± 0.20	0.411 ± 0.21	0.260 ± 0.15
Bayesian (particle filter)	0.386 ± 0.17	0.370 ± 0.17	0.259 ± 0.14
t-RNN	0.372 ± 0.17	0.345 ± 0.17	0.251 ± 0.13
d-RNN	0.349 ± 0.15	0.326 ± 0.16	0.243 ± 0.13

References

1. Dezfouli A, Griffiths K, Ramos F, Dayan P, Balleine BW. Models that learn how humans learn: the case of decision-making and its disorders. PLoS computational biology. 2019;15(6):e1006903.
