# Supplementary material for: Harnessing the flexibility of neural networks to predict dynamic theoretical parameters underlying human choice behavior
Source: PLoS Comput Biol. 2024 Jan 4;20(1):e1011678. doi: 10.1371/journal.pcbi.1011678 (PMC10793919; doi:10.1371/journal.pcbi.1011678)
Supplement: S5 Table — Summary statistics of the trial-by-trial RL parameter estimation produced by t-RNN for each diagnostic group. (PDF) [file pcbi.1011678.s006.pdf]

Summary statistics of RL parameters estimations by diagnostic group. Summary statistics of the trial-by-trial RL parameter estimation produced by t-RNN for each diagnostic group.

Table S5. RL parameter estimation for each diagnostic group. Behavioral dataset from [1]. Mean ± SD.

**Diagnostic group**	α	β	κ
Bipolar $N = 33$	0.331±0.12	0.028±0.01	0.021±0.01
Depression $N = 34$	0.330±0.12	0.022±0.01	0.020±0.01
Healthy $N = 34$	0.332±0.12	0.025±0.01	0.020±0.01

References

1. Dezfouli A, Griffiths K, Ramos F, Dayan P, Balleine BW. Models that learn how humans learn: the case of decision-making and its disorders. PLoS computational biology. 2019;15(6):e1006903.
