# Supplementary material for: Harnessing the flexibility of neural networks to predict dynamic theoretical parameters underlying human choice behavior
Source: PLoS Comput Biol. 2024 Jan 4;20(1):e1011678. doi: 10.1371/journal.pcbi.1011678 (PMC10793919; doi:10.1371/journal.pcbi.1011678)
Supplement: S6 Table — Summary table of the raw results presented in the main text (see Fig 4A). (PDF) [file pcbi.1011678.s007.pdf]

Action prediction behavioral dataset [1]. Summary table of the raw results presented in the main text (see Fig 4A).

Table S6. Action prediction for each model. Behavioral dataset from [1]. Measured with binary cross-entropy. ↓ lower is better. $N = 44$. Mean ± SD.

**Model**	BCE ↓
Stationary hybrid explore.	0.331 ± 0.07
Bayesian (particle filter)	0.332 ± 0.07
t-RNN	0.320 ± 0.07
d-RNN	0.310 ± 0.08

References

1. Gershman SJ. Deconstructing the human algorithms for exploration. Cognition. 2018;173:34–42.
