# Supplementary material for: Harnessing the flexibility of neural networks to predict dynamic theoretical parameters underlying human choice behavior
Source: PLoS Comput Biol. 2024 Jan 4;20(1):e1011678. doi: 10.1371/journal.pcbi.1011678 (PMC10793919; doi:10.1371/journal.pcbi.1011678)
Supplement: S1 Fig — The left panels show the trial-by-trial RL κ perseveration parameter estimation using t-RNN (blue) and QP-stationarity (green) methods, along with the moving average calculation of the stay probabilities (red; window size of 10 trials) for three example subjects (one from each diagnostic group). The right panels show the corresponding Pearson correlation between the moving average stay probabilities and κ parameter estimation of t-RNN and QP-stationarity model (red dashed line denotes the identity). The results indicate a strong correlation between the stay probabilities and κ parameter estimation of t-RNN (r2 > 0.9), but not of the QP-stationarity (r ≈ 0), which fails to detect changes in subject behavior throughout the task. (PDF) [file pcbi.1011678.s008.pdf]

Relation of perseveration estimation with stay probability. Further details examples of the analysis presented in the main text (see Fig. 3C), in which we computed a Pearson correlation between the moving average stay probabilities and the t-RNN trial-by-trial κ perseveration estimation.

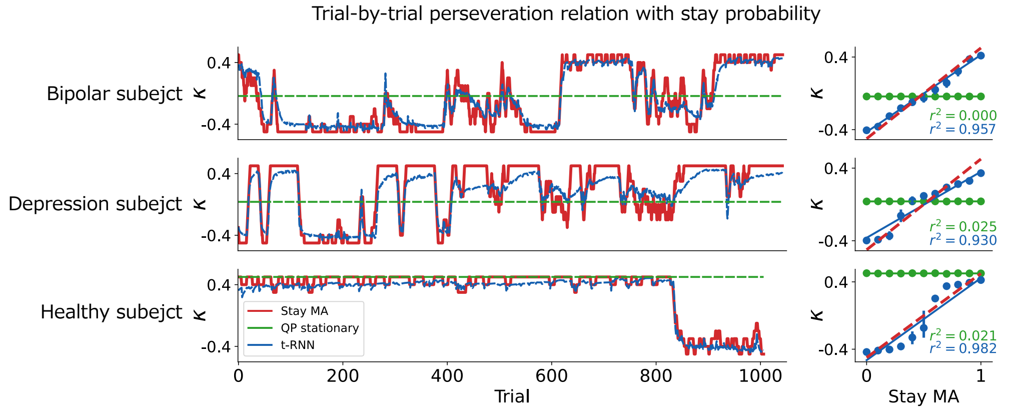

Fig S1. The left panels show the trial-by-trial RL κ perseveration parameter estimation using t-RNN (blue) and QP-stationarity (green) methods, along with the moving average calculation of the stay probabilities (red; window size of 10 trials) for three example subjects (one from each diagnostic group). The right panels show the corresponding Pearson correlation between the moving average stay probabilities and κ parameter estimation of t-RNN and QP-stationarity model (red dashed line denotes the identity). The results indicate a strong correlation between the stay probabilities and κ parameter estimation of t-RNN ($r^2 > 0.9$), but not of the QP-stationarity ($r \approx 0$), which fails to detect changes in subject behavior throughout the task.
