# Supplementary material for: Harnessing the flexibility of neural networks to predict dynamic theoretical parameters underlying human choice behavior
Source: PLoS Comput Biol. 2024 Jan 4;20(1):e1011678. doi: 10.1371/journal.pcbi.1011678 (PMC10793919; doi:10.1371/journal.pcbi.1011678)
Supplement: S2 Fig — (A) Boxplot of the time-varying β estimates for each diagnostic group (middle black solid lines denote the median; light dots indicate a single trial estimate of the κ preservation parameter). Results indicate that all groups exhibit similar estimates, with a slight increase in the healthy group compared to the clinical groups. This suggests that the healthy group’s action selection was slightly less random than the clinical group’s. (B) Trial-by-trial β inverse-temperature parameter estimates by t-RNN averaged over the first 100 trials of each block and for each diagnostic group separately (shaded area signifies the s.e.m). In all three groups, subjects show an increase in their β estimates (behavior became less random as the block progressed), but the baseline differs, with clinical groups having a lower starting point. (C) Distribution of the Pearson correlation between the choice probabilities produced by t-RNN (calculated using a moving average of 10 trials) and the time-varying β inverse-temperature produced by t-RNN for each subject individually. The results indicate a strong relationship between t-RNN time-varying RL β parameter estimation and the moving average of the choice probabilities. (PDF) [file pcbi.1011678.s009.pdf]

**Additional analysis inverse-temperature estimation.** In the main text, our analysis mainly focused on the dynamical $\kappa$ perseveration parameter estimation produced by t-RNN. For completeness, we provide here a similar analysis for the $\beta$ inverse-temperature which determines the randomness of action selection. To further support the interpretability of t-RNN parameters, in Fig S2C we used another easy-to-interpret model-agnostic measurement. We calculated a moving average of the distance between t-RNN action prediction probabilities and the probabilities of random choice policy (i.e., $|p_{tRNN}(a_t) - 0.5|$; window size of 10 trials). If t-RNN $\beta$ estimates are related to the degree of randomness in action selection, we should expect a high correlation with the moving average model-agnostic estimate. We calculated a Pearson correlation between the moving average and t-RNN trial-by-trial $\beta$ estimations for each individual. Overall, we found that the median Pearson correlation across individuals was very high (Median=0.74).

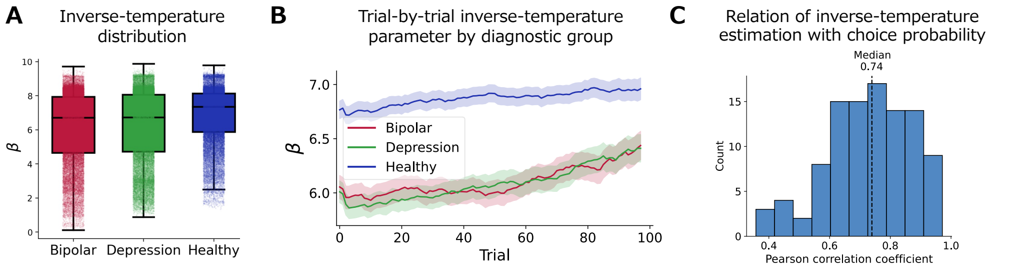


**Fig S2. Analysis of the t-RNN trial-by-trial $\beta$ inverse-temperature estimation. (A)** Boxplot of the time-varying $\beta$ estimates for each diagnostic group (middle black solid lines denote the median; light dots indicate a single trial estimate of the $\kappa$ preservation parameter). Results indicate that all groups exhibit similar estimates, with a slight increase in the healthy group compared to the clinical groups. This suggests that the healthy group's action selection was slightly less random than the clinical group's. **(B)** Trial-by-trial $\beta$ inverse-temperature parameter estimates by t-RNN averaged over the first 100 trials of each block and for each diagnostic group separately (shaded area signifies the s.e.m). In all three groups, subjects show an increase in their $\beta$ estimates (behavior became less random as the block progressed), but the baseline differs, with clinical groups having a lower starting point. **(C)** Distribution of the Pearson correlation between the choice probabilities produced by t-RNN (calculated using a moving average of 10 trials) and the time-varying $\beta$ inverse-temperature produced by t-RNN for each subject individually. The results indicate a strong relationship between t-RNN time-varying RL $\beta$ parameter estimation and the moving average of the choice probabilities.
