# Supplementary material for: Harnessing the flexibility of neural networks to predict dynamic theoretical parameters underlying human choice behavior
Source: PLoS Comput Biol. 2024 Jan 4;20(1):e1011678. doi: 10.1371/journal.pcbi.1011678 (PMC10793919; doi:10.1371/journal.pcbi.1011678)
Supplement: S3 Fig — The left panels show the trial-by-trial RL β parameter estimation of t-RNN (blue; divided by 20 to fit the scale) and a moving average calculation of the absolute value difference between the choice probabilities produced by t-RNN and random choice probability (red; window size of 10 trials) for three example subjects (one from each diagnostic group). The right panels show the corresponding Pearson correlation between the moving average choice probabilities and β parameter estimation of t-RNN (red dashed line denotes the identity). The results indicate a strong correlation between the two estimations. (PDF) [file pcbi.1011678.s010.pdf]

Relation of inverse-temperature estimation with random choice. To illustrate our analysis approach, as shown in Fig S2C, we showcase the results of three example subjects, one from each diagnostic group.

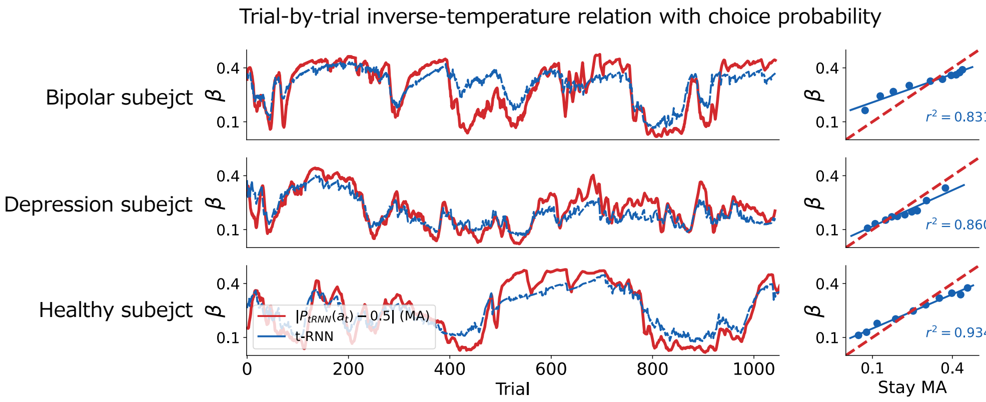

Fig S3. The left panels show the trial-by-trial RL β parameter estimation of t-RNN (blue; divided by 20 to fit the scale) and a moving average calculation of the absolute value difference between the choice probabilities produced by t-RNN and random choice probability (red; window size of 10 trials) for three example subjects (one from each diagnostic group). The right panels show the corresponding Pearson correlation between the moving average choice probabilities and β parameter estimation of t-RNN (red dashed line denotes the identity). The results indicate a strong correlation between the two estimations.
