# Supplementary material for: Harnessing the flexibility of neural networks to predict dynamic theoretical parameters underlying human choice behavior
Source: PLoS Comput Biol. 2024 Jan 4;20(1):e1011678. doi: 10.1371/journal.pcbi.1011678 (PMC10793919; doi:10.1371/journal.pcbi.1011678)
Supplement: S4 Fig — In Fig 3D we depicted specific trials to illustrate the estimation of parameters across the two methods (QP-stationary, t-RNN). For completeness, we present the full range of trials for the same subjects (black boxes represent the trials that are depicted in Fig 3D). (A) Example bipolar subject. (B) Example depression subject. Action prediction (top) and theoretical RL κ parameters estimation (bottom). (PDF) [file pcbi.1011678.s011.pdf]

Supplement Fig. 3D We present here the full trial trajectories and model predictions of the two subjects presented in Fig. 3D.

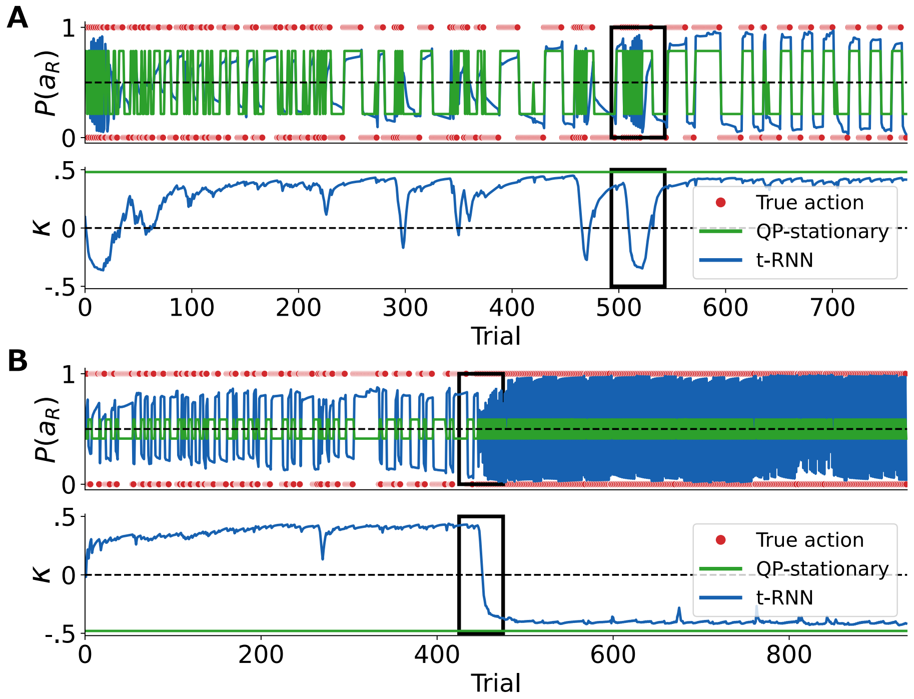

Fig S4. Supplement Fig 3D. In Fig. 3D we depicted specific trials to illustrate the estimation of parameters across the two methods (QP-stationary, t-RNN). For completeness, we present the full range of trials for the same subjects (black boxes represent the trials that are depicted in Fig. 3D). **(A)** Example bipolar subject. **(B)** Example depression subject. Action prediction (top) and theoretical RL κ parameters estimation (bottom).
