# Supplementary material for: Harnessing the flexibility of neural networks to predict dynamic theoretical parameters underlying human choice behavior
Source: PLoS Comput Biol. 2024 Jan 4;20(1):e1011678. doi: 10.1371/journal.pcbi.1011678 (PMC10793919; doi:10.1371/journal.pcbi.1011678)
Supplement: S5 Fig — (A) Example of a single instantiation of a single action block. The top panel depicts the sequence of chosen actions, obtain rewards, and t-RNN action predictions as red dots, red numbers, and blue solid lines, respectively. The subject consecutively chooses the same action. The bottom panel depicts t-RNN γ directed exploration parameter estimation, where it estimates a step decrease in the parameter throughout the block. (B) Dynamics of the directed exploration parameter averaged across all instantiations of a single action block. t-RNN estimates a sharp decrease in the γ directed exploration parameter in these blocks. (PDF) [file pcbi.1011678.s012.pdf]

Directed exploration dynamics of repeated action blocks. We conducted an additional investigation into the trial-by-trial dynamics of the t-RNN theoretical parameter estimation of the behavioral dataset [1]. Specifically, we identified a block of 10 consecutive trials where an individual persisted in choosing the same option throughout. Consistent with our expectations, we observed a significant reduction in the t-RNN's γ parameter estimation of directed exploration, indicating a lack of interest in exploring the less familiar option (see Fig S5A). To ascertain whether this pattern holds true across individuals and trials, we aggregated the t-RNN's γ parameter estimates over all blocks (a total of 70 blocks) where individuals chose the same option. Our findings revealed a similar trend of a sharp decrease in the γ directed exploration parameter estimation (mean $\pm$ s.e.m. before: $0.52 \pm .02$, after: 0.18 ± 0.01).

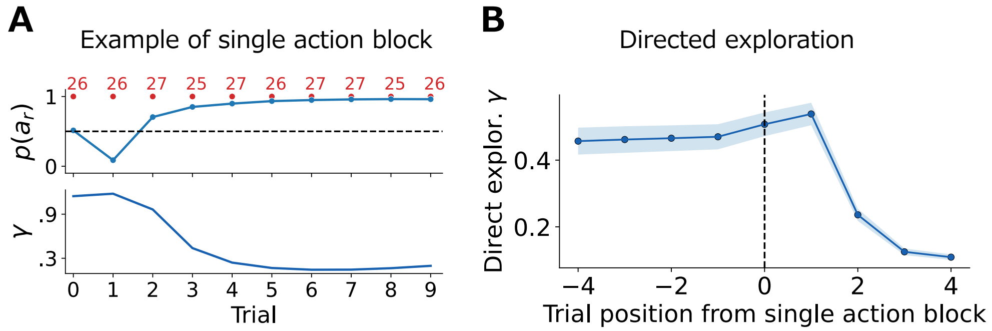

Fig S5. (A) Example of a single instantiation of a single action block. The top panel depicts the sequence of chosen actions, obtain rewards, and t-RNN action predictions as red dots, red numbers, and blue solid lines, respectively. The subject consecutively chooses the same action. The bottom panel depicts t-RNN γ directed exploration parameter estimation, where it estimates a step decrease in the parameter throughout the block. **(B)** Dynamics of the directed exploration parameter averaged across all instantiations of a single action block. t-RNN estimates a sharp decrease in the γ directed exploration parameter in these blocks.

References

1. Gershman SJ. Deconstructing the human algorithms for exploration. Cognition. 2018;173:34–42.
