# Supplementary material for: Harnessing the flexibility of neural networks to predict dynamic theoretical parameters underlying human choice behavior
Source: PLoS Comput Biol. 2024 Jan 4;20(1):e1011678. doi: 10.1371/journal.pcbi.1011678 (PMC10793919; doi:10.1371/journal.pcbi.1011678)
Supplement: S6 Fig — Training t-RNN without concatenation (green bar) led to a degradation in the performance in the action prediction compared to t-RNN trained with concatenation (blue bar) for both empirical datasets (error measured in BCE; black lines indicate s.e.m; *** p < .001; ** p < .01). (PDF) [file pcbi.1011678.s013.pdf]

t-RNN concatenation ablation. We conducted an ablation analysis in which we trained a t-RNN model without concatenation of the categorical class predictions of the RL parameters into the action prediction head, and compared it to the original method that was trained with concatenation. Results revealed that training t-RNN without concatenation led to a degradation in the performance in the action prediction for both empirical datasets tested (Wilcoxon signed rank test; *** $p < .001$ for Dezfouli et al. dataset [1]; ** $p < .01$ for Gershman dataset [2]).

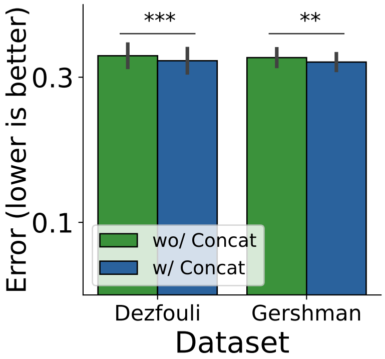

Fig S6. t-RNN concatenation ablation. Training t-RNN without concatenation (green bar) led to a degradation in the performance in the action prediction compared to t-RNN trained with concatenation (blue bar) for both empirical datasets (error measured in BCE; black lines indicate s.e.m; *** $p < .001$; ** $p < .01$)

References

1. Dezfouli A, Griffiths K, Ramos F, Dayan P, Balleine BW. Models that learn how humans learn: the case of decision-making and its disorders. PLoS computational biology. 2019;15(6):e1006903.

2. Gershman SJ. Deconstructing the human algorithms for exploration. Cognition. 2018;173:34–42.
