# Supplementary material for: Harnessing the flexibility of neural networks to predict dynamic theoretical parameters underlying human choice behavior
Source: PLoS Comput Biol. 2024 Jan 4;20(1):e1011678. doi: 10.1371/journal.pcbi.1011678 (PMC10793919; doi:10.1371/journal.pcbi.1011678)
Supplement: S7 Fig — (a) alpha parameter estimation, (b) beta parameter estimation. The top panel represents recovery of stationary RL parameters, the middle depicts recovery of abruptly changing RL parameters, and the bottom shows recovery of gradually changing RL parameters. Ground truth (red), stationary Q-learning (green), Bayesian (yellow), our t-RNN with static training set (pink), and t-RNN (blue). Error bars, Bayesian model (in yellow; calculated using the variance over the particles). Error bars, ours (in blue; calculated using 10 different runs of our model). (PDF) [file pcbi.1011678.s014.pdf]

Using ensemble method to gain variance estimation for t-RNN. We wanted to examine on a provisional basis whether variance estimation can be obtained for t–RNN by using an ensemble method. For this aim, we trained ten different networks and then used the summary statistics (mean/sd) as trial-by-trial latent parameter estimation. Error bars for the Bayesian model were calculated using the variance in the particles. It can be observed in Fig S7 that the epochs with increased error bars for t-RNN somewhat overlap with those that show an increase in error bars for the Bayesian particle filtering (e.g., top left panel describing results for stationary alpha trials 200-300, also middle left panel describing results for abruptly changing alpha trials 800-900). However, further studies are needed to directly and comprehensively compare different methods for obtaining variance estimation for t-RNN, and understand how they can be applied to empirical data.

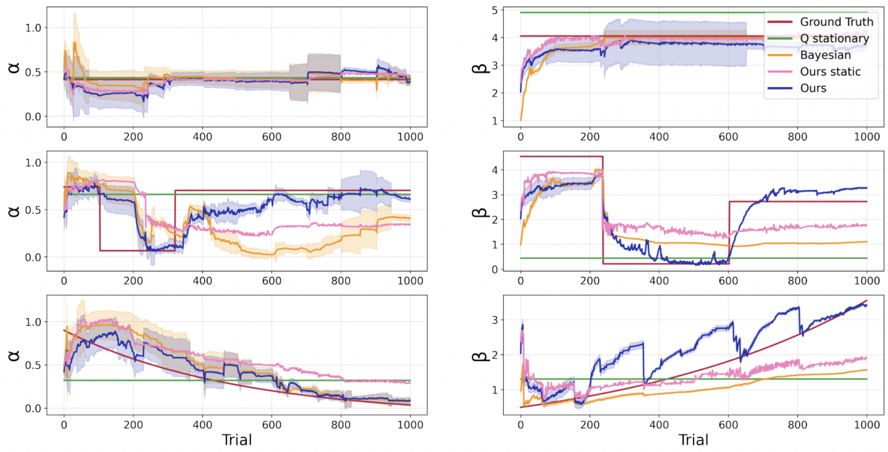

(a) (b)

Fig S7. Using ensemble method to gain variance estimation. (a) alpha parameter estimation, (b) beta parameter estimation. The top panel represents recovery of stationary RL parameters, the middle depicts recovery of abruptly changing RL parameters, and the bottom shows recovery of gradually changing RL parameters. Ground truth (red), stationary Q-learning (green), Bayesian (yellow), our t-RNN with static training set (pink), and t-RNN (blue). Error bars, Bayesian model (in yellow; calculated using the variance over the particles). Error bars, ours (in blue; calculated using 10 different runs of our model).
